# Magnetic Resonance-Guided Radiation Therapy for Head and Neck Cancers

**DOI:** 10.3390/curroncol29110655

**Published:** 2022-10-31

**Authors:** Danny Lavigne, Sweet Ping Ng, Brian O’Sullivan, Phuc Felix Nguyen-Tan, Edith Filion, Laurent Létourneau-Guillon, Clifton D. Fuller, Houda Bahig

**Affiliations:** 1 Department of Radiation Oncology, Centre Hospitalier de l’Université de Montréal, University of Montreal, Montreal, QC H2X 3E4, Canada; 2 Department of Radiation Oncology, Olivia Newton-John Cancer Centre, Austin Health, Melbourne, VI 3084, Australia; 3 Department of Radiology, Centre Hospitalier de l’Université de Montréal, University of Montreal, Montreal, QC H2X 3E4, Canada; 4 Department of Radiation Oncology, MD Anderson Cancer Center, University of Texas, Houston, TX 77030, USA

**Keywords:** radiation therapy, head and neck cancer, magnetic resonance imaging, magnetic resonance-guided radiation therapy, MR-Linac

## Abstract

Despite the significant evolution of radiation therapy (RT) techniques in recent years, many patients with head and neck cancer still experience significant toxicities during and after treatments. The increased soft tissue contrast and functional sequences of magnetic resonance imaging (MRI) are particularly attractive in head and neck cancer and have led to the increasing development of magnetic resonance-guided RT (MRgRT). This approach refers to the inclusion of the additional information acquired from a diagnostic or planning MRI in radiation treatment planning, and now extends to online high-quality daily imaging generated by the recently developed MR-Linac. MRgRT holds numerous potentials, including enhanced baseline and planning evaluations, anatomical and functional treatment adaptation, potential for hypofractionation, and multiparametric assessment of response. This article offers a structured review of the current literature on these established and upcoming roles of MRI for patients with head and neck cancer undergoing RT.

## 1. Introduction

Radiation therapy (RT) plays a leading role in the definitive and postoperative management of head and neck cancers, with most patients undergoing 6 to 7 weeks of conventionally fractionated daily treatments [1]. The parallel advancements in imaging modalities and in radiation planning and delivery techniques, including 3-dimensional conformal RT (3D-CRT), intensity-modulated RT (IMRT) and image-guided RT (IGRT), allowed for progressively more precise and less toxic treatments for patients undergoing RT [2,3]. Although these innovations were largely made possible by the development and integration of computed tomography (CT) and cone beam CT, their limited quality for the visualization of soft tissues is suboptimal for tumors originating from the head and neck region. Magnetic resonance imaging (MRI), on the other hand, differentiates and describes soft tissues more easily and accurately, and has the advantage of providing functional data regarding the tumor and normal tissues. Moreover, in contrast to CT, MRI does not rely on ionizing radiation, allowing for safer repeated imaging as needed. For these reasons, a diagnostic or planning MRI co-registered with the planning CT is often useful during radiation treatment planning for head and neck cancers [4]. These advantages were also the basis for the development of one of the latest technological innovations in radiation oncology: the MR-Linac, which combines a radiation treatment unit with a high-resolution MRI for daily imaging [5]. This novel technology broadens the horizon to a wider use of magnetic resonance-guided RT (MRgRT). The current literature investigating the benefits of the MR-Linac is mainly focused on genitourinary and gastrointestinal cancers, aiming to reduce the impact of mobile organs such as the bowel and bladder on RT delivery. Although head and neck cancer patients are subjected to a lesser degree of inter- and intra-fraction motion, the immediate proximity of their disease with many vulnerable soft tissues makes this innovative technology and MRgRT very attractive for this population.

The increasing use of MRI for head and neck cancers RT in the recent years was followed by a proportional increase in the publication rate on the subject. However, very few reviews synthesizing the current use of MRgRT have been published, and none overviewed the many roles of MRI in head and neck cancers RT. This review describes the current and upcoming roles of MRI for head and neck cancer patients undergoing RT, including baseline diagnostic and planning evaluation, treatment adaptation, and assessment of response to therapy, while also presenting the current challenges limiting its broader use.

## 2. Baseline Evaluation

### 2.1. Local Extension

The MRI’s ability to distinguish and describe normal and abnormal soft tissues has become a great asset for the diagnosis and staging of head and neck cancers and is now part of the standard of care for many patients. The National Comprehensive Cancer Network (NCCN)’s guidelines recommend an MRI over a CT during the initial workup for various clinical scenarios, including suspicion of bone marrow involvement, presence of dental amalgams, nasopharyngeal cancers, sinonasal cancers and any head and neck cancer with suspected perineural invasion (PNI) [1].

**Figure 1:**
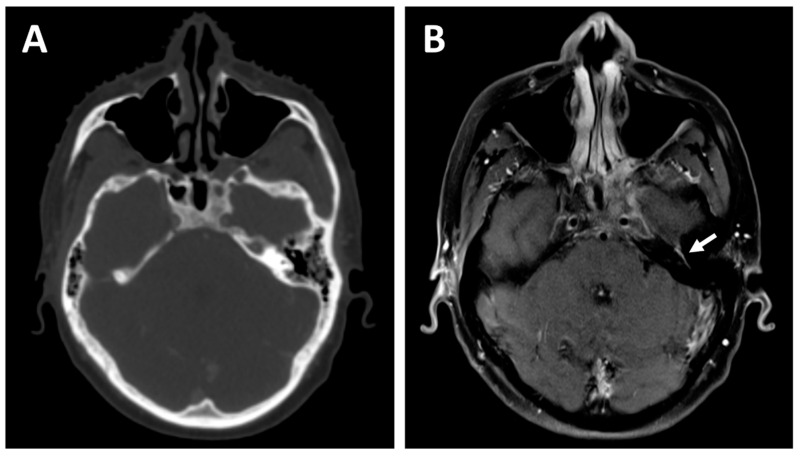
A 27-year-old patient with a cT4N2 undifferentiated carcinoma of the nasopharynx who received 3 cycles of Cisplatin and Gemcitabine induction chemotherapy before concurrent chemoradiotherapy. The planning contrast-enhanced computed tomography (CT) (**A**) demonstrates excellent response to the induction treatment but fails to detect residual perineural involvement of the left facial nerve, visualized as an asymmetrical enhancement on the planning T1-weighted contrast-enhanced magnetic resonance imaging (MRI) (**B**)—white arrow. The facial nerve involvement could be tracked down to the left parotid and was consequently included in the radiation treatment volume.

**Figure 2:**
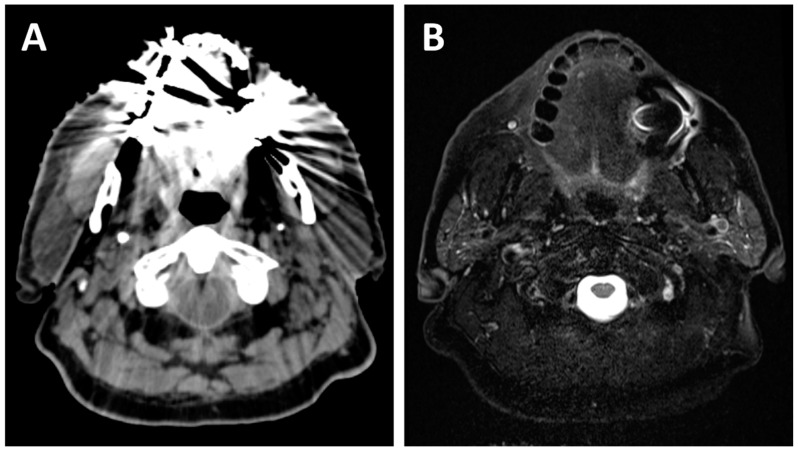
A 70-year-old patient with dental amalgam planned for concurrent chemoradiotherapy for a cT3N2b squamous cell carcinoma of the right base of tongue. The planning CT (**A**) shows poor image quality with important artifacts which could easily obscure oral or oropharyngeal pathology. The planning T2-weighted MRI (**B**) also shows artifacts due to dental amalgam, but the image quality remains sufficient for adequate radiological evaluation of most of the oral cavity and oropharynx.

The first and probably most important advantage of MRI for head and neck cancers is its high soft tissue contrast, allowing for better anatomic description of tumors and the potential subsites which they invade [6]. This allows for better delineation of the margins of the disease and provides a more sensitive evaluation of vascular and perineural invasion, intracranial extension, and bone marrow involvement [7]. This is particularly important for head and neck cancers, as the size and anatomic subsites invaded correspond to the tumor stage and directly impact the treatment options and extent of radiation coverage. As many major nerves surround the head and neck structures, cancers in this region may also spread along them, which has been shown to be associated with a worse prognosis and higher rates of recurrences [8]. Some histological tumor subtypes originating from the head and neck region are known to be at higher risk of PNI, including adenoid cystic carcinomas and cutaneous squamous cell carcinomas. MRI has a higher sensitivity and specificity for the detection of PNI when compared to CT [9] or fluorodeoxyglucose-positron emission tomography (FDG-PET) [10], and is therefore the modality of choice for patients susceptible to this risk or in whom PNI is suspected. Figure 1 depicts an example of a patient with a nasopharyngeal carcinoma with PNI of the left facial nerve detected on a radiation treatment planning MRI. Furthermore, MRI has a higher sensitivity and specificity than FDG-PET or CT for muscle invasion and seems to correlate the lesion size to the histopathologic specimen more accurately [10]. In contrast to CT, MRI also has the benefit of being less affected by dental restorations or densely calcified structures, as visualized on Figure 2, which can have an important impact on its diagnostic abilities for the evaluation of oropharyngeal and oral cancers [11].

### 2.2. Lymph Node Evaluation

Although CT is usually the first imaging examination used to identify metastatic cervical lymph nodes in head and neck cancers, its criteria for malignancy are mostly based on size, shape, contours, and number [12]. Smaller lymph nodes seen on CT are often described as non-specific, but when RT treatments are planned, it becomes hard to determine how to approach these subcentimeter nodes. FDG-PET can sometimes be helpful in such cases, with a high specificity for metastatic lymph nodes, but its sensitivity appears to be as low as 31% for subcentimeter nodes compared to 90% for nodes larger than 1 cm [13]. Although not yet standard in clinical practice, functional MRI techniques including diffusion-weighted imaging (DWI) [14], 3D dynamic contrast-enhanced (DCE) [15], spectroscopy [16] and perfusion MRI have shown promising results for differentiating malignant and benign cervical lymph nodes. DWI, which measures the restricted water diffusion in tissues, has consistently shown lower apparent diffusion coefficient (ADC) in metastatic lymph nodes compared to benign lymph nodes [17]. Several groups have reported a high sensitivity and specificity for the detection of metastatic cervical lymph nodes with DWI, ranging from 52 to 100% and 84 to 97%, respectively [17]. One prospective study focusing on subcentimeter cervical nodes found a sensitivity of 88% and a specificity of 81% when using a smaller region of interest (ROI) placed in the middle of the node for the measurement of ADC, compared to 80% when using the whole node as the ROI [18]. As for the detection of extranodal extension, which is associated with an adverse prognosis for patients with head and neck cancers, two meta-analyses showed no significant difference between MRI and CT in terms of sensitivity and specificity [19,20].

**Figure 3:**
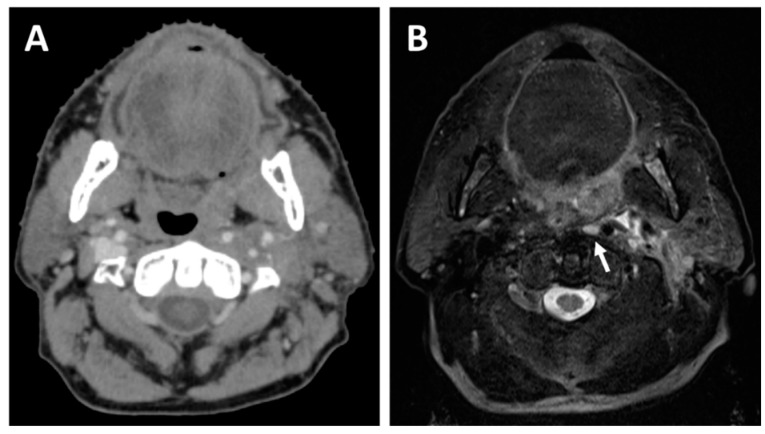
A 64-year-old patient with a cT2N1 p16-positive squamous cell carcinoma of the left tonsil planned for concurrent chemoradiotherapy. The planning CT (**A**) shows a mass effect originating from the left tonsil, whereas the planning T2-weighted MRI (**B**) allows better visualization of the left tonsil tumor and also detects a left retropharyngeal lymph node metastasis (white arrow).

Another advantage of MRI during baseline lymph node evaluation consists of its superior detection of retropharyngeal lymph node (RPLN) metastases. CT has been shown to be insufficient for the detection of metastases in the RPLN, which are located between the pharyngeal constrictor muscles and the longus capitis and longus colli muscles, medial to the carotid arteries [21]. This deep region is not easily accessible surgically, and its assessment therefore relies on imaging modalities. A comparative study of patients with histologically confirmed RPLN metastases revealed that MRI has a higher sensitivity (74%) than CT (65%) for their radiological detection, and the combination of CT and MRI with an FDG-PET further increased the sensitivity (90%) [22]. Figure 3 shows an example of a left retropharyngeal lymph node metastasis better seen on a planning MRI compared to CT. As RPLN metastases are known to have a detrimental effect on prognosis, and since their detection impacts treatment decisions, this highlights the importance of a multimodality imaging evaluation for head and neck cancer patients [23,24,25].

### 2.3. Treatment Planning

Most RT treatment courses for head and neck cancers comprise at least two levels of irradiation dose: a higher dose encompassing the gross tumor volume (GTV), and a lower dose covering areas of potential microscopic disease. As the GTV is directly based on the delineation of the macroscopic disease, its adequate visualization during treatment planning is of utmost importance [26]. The superiority of MRI for describing the local extension of head and neck cancers into adjacent anatomic subsites, muscles, and nerves is therefore a great asset during the planning of precise RT treatments. In fact, the Danish Head and Neck Cancer Group (DAHANCA)’s 2020 radiotherapy guidelines recommend the use of multimodality imaging including an MRI during treatment planning for all patients with a macroscopic tumor in place and for postoperative cases to compare with the preoperative MRI and to benefit from its superior soft tissue contrast to delineate the tumor bed [4,27]. The international head and neck consensus guidelines published in 2015 also strongly recommend the use of an MRI during RT planning for primary tumors of the nasopharynx, oral cavity, and oropharynx, and for the delineation of several organs at risk (brainstem, spinal cord, pituitary gland, lacrimal glands, optic chiasm, and optic nerves) [28].

**Figure 4:**
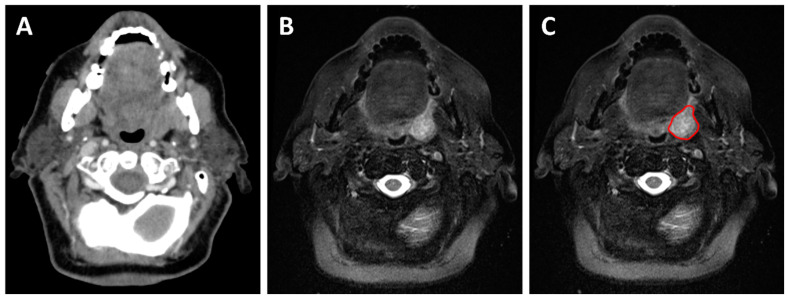
A 73-year-old patient with a cT2N1 p16-positive squamous cell carcinoma of the left tonsil planned for concurrent chemoradiotherapy. The planning contrast-enhanced CT (**A**) shows an asymmetrical left tonsil with poorly defined margins. The planning T2-weighted MRI (**B**,**C**) allows better visualization of the tumor margin and aids in the delineation of the gross tumor volume during radiation treatment planning (red contour).

Specific cases where a pre-treatment MRI substantially affects treatment planning include nasopharyngeal cancers, where MRI was shown to significantly increase the rate of detection of intracranial and pterygopalatine fossa invasion compared to CT [29]. For sinonasal cancers, MRI also facilitates the evaluation of dural, intracranial and orbital invasion [17]. These findings have a direct impact on volume delineation. The addition of an MRI for the delineation of base of tongue cancers also showed a significant increase in the gross tumor volume compared to CT delineation, suggesting that MRI detects parts of the tumor not seen on CT [30]. The same study also revealed smaller volumes for the brainstem and spinal cord and improved delineation of parotid glands, due to improved organ visualization on MRI [30]. Figure 4 depicts the added value of an MRI during the radiation treatment planning of a patient with a cT2N2 squamous cell carcinoma of the left tonsil. Moreover, the presence of perineural spread which, as previously mentioned, is more precisely assessed on MRI, may warrant the extension of RT volumes to cover probable routes of spread along the nerves to reduce the risk of disease recurrence [8]. A sensitive detection of RPLN metastases is also essential to optimize treatment planning, as involved RPLN will be included within the high-risk target volume receiving the highest radiation dose [31]. An accurate understanding of local disease extension is also necessary to correctly define the low-risk target volume, which depends on the tumor stage and on the anatomic subsites invaded. For example, a clinical T3 or T4 nasopharyngeal carcinoma would warrant coverage of the cavernous sinus, in contrast to T1 and T2 tumors where it could usually be omitted [32]. The low-risk target volume also covers the neck node levels at risk of microscopic disease, and the choice of the specific levels to include also depends on the anatomic subsites involved [31]. 

One limitation of MRI-based delineation is a potentially high interobserver variability, as was described in the published abstract of the International MRI Linear Accelerator Oropharyngeal Carcinoma Delineation Study [33]. However, the addition of a PET and CT to MRI did not improve this variability in their study. In contrast, another group found a reduction in interobserver variation for target delineation in nasopharyngeal cancers when the physicians were provided contouring instructions and a co-registered MRI [34]. These conflicting results mostly highlight the need for international MRI-based contouring guidelines.

## 3. Treatment Adaptation

### 3.1. CT-Based Treatment Adaptation

As head and neck cancer patients undergo their 6 to 7 weeks of daily radiation treatments, weight loss and tumor response often lead to notable anatomical changes. These changes may lead to the migration of organs at risk such as the parotids and pharyngeal constrictor muscles to high dose regions, resulting in an unplanned overdosage and potentially worse predicted chronic xerostomia [35,36,37]. Intended target volumes may also be at risk of inadequate coverage, possibly jeopardizing local control [38]. In a small prospective trial evaluating the need for replanning at weeks 3 and 6 during the course of RT, CT-based adaptive RT reduced doses to the spinal cord, avoided overdosage of the parotids and improved target coverage and homogeneity [39]. A second prospective trial by Schwartz et al. confirmed the reduction in mean parotid dose with CT-based adaptive RT [40]. One properly timed replanning seemed to deliver most of these dosimetric improvements. CT-based replanning is now standard for head and neck cancers and is usually done once the anatomical changes noted on the daily cone-beam CT seem important enough to significantly alter the initial dosimetry.

### 3.2. MRI-Based Treatment Adaptation

#### 3.2.1. Anatomical Adaptation

Although MRI has now been widely adopted during the treatment planning of many head and neck cancers, its superiority in describing local extension and nodal disease has potential uses for RT beyond initial baseline evaluation, notably the opportunity for adaptive planning.

For its many aforementioned advantages in the head and neck region, repeated MRI during the course of radiotherapy could further improve treatment adaptation by allowing better visualization of the tumor response and optimal target volume delineation. Subesinghe et al. reported a significant tumor shrinkage as early as by the 11th treatment fraction for head and neck cancer patients treated with RT, while Ding et al. found a complete response rate as high as 50% by mid-treatment for patients with human papillomavirus (HPV)-related oropharyngeal cancer (OPC) [41,42]. The latter study also demonstrated that the volume change happens early for the primary tumor, whereas the trajectory of nodal disease shrinkage is more linear [42]. Such important tumor responses, especially early during the treatment course, raise the question of whether maintaining the initially planned GTV is necessary, or if these early-responding patients are being overtreated with a consequent unnecessary risk of toxicity. The accuracy of the initial dosimetry plan may also be questioned given the anatomical changes. In that sense, a prospective in silico study from the MD Anderson MRLinac Development Working Group showed that response-based biweekly MR-guided adaptive RT for HPV-related OPC patients could reduce the probability of chronic dysphagia by 11% and the mean parotid dose by 3.3 Gy in comparison to standard IMRT. They also showed that the GTV volume decreased on average by 44, 90 and 100% at weeks 2, 4 and 6, respectively, while the nodal volumes decreased by 25, 60 and 80% during the same period [43].

Although these results are very promising for the reduction of toxicities for these patients, randomized data are needed to prove the safety and clinical benefit of MRI-based adaptive RT. In particular, the impact on local control of reducing target volumes to only the shrinking MRI-visible disease must be assessed, since tumors could potentially be dissolving instead of shrinking, leaving behind microscopic disease in areas previously occupied by tumor. Fortunately, several prospective randomized and single-arm clinical trials investigating the benefit and safety of MRgRT are currently ongoing. MR-ADAPTOR is an R-IDEAL stage 2a–2b/Bayesian phase II trial led by the MD Anderson Cancer Center. The first stage of the trial will enroll 15 low-risk HPV-positive OPC patients to MRgRT dose adaptation with a weekly MRI-based RT replan before enrolling 60 additional patients randomized to either MRgRT or standard IMRT, with their primary outcome being locoregional control (LRC) [44]. ART-OPC is another phase II randomized trial currently recruiting and aiming to enroll 120 patients with advanced OPC who will be randomized to either standard IMRT or MRgRT with a single MRI-based RT replan on week 3 of treatments. This trial is assessing the rates of chronic dysphagia, toxicities, LRC, overall survival (OS) and quality of life (QoL) associated with this novel technique [45]. The MARTHA trial is a phase II single arm trial recruiting patients with locally advanced head and neck cancers to assess the rate of xerostomia associated with daily imaging for MR-IGRT and once weekly offline plan adaptation [46]. The INSIGHT-2 trial is a phase I/II single arm trial also recruiting advanced head and neck cancer patients, aiming to assess the feasibility and safety of MR-guided ART at weeks 2 and 4 and, for HPV-negative patients, dose escalation at week 2 according to response identified on DWI [47].

#### 3.2.2. Functional Adaptation

This last trial highlights another potential role for MRI in adaptive planning beyond the assessment of anatomical changes during treatments, which is the opportunity for functional adaptation. It is now well known that head and neck cancers are widely heterogeneous with an obvious example being HPV-associated OPC, which is associated with a significantly better prognosis and a greater response to therapy [48]. For this reason, in the past decade, much effort has extended towards tailoring treatments to individual patients to enhance control of more resistant tumors (treatment escalation), and to reduce treatment toxicities for patients with better responding tumors (treatment de-escalation). DWI-guided treatment adaptation was also investigated in a phase II randomized clinical trial for locoregionally advanced nasopharyngeal cancers, where patients received either standard IMRT or dose-painted dose-escalation to tumor regions showing lower ADC on DWI. This experimental technique showed promising results with an improved 2-year disease-free survival of 93.6% vs. 87.5% for standard IMRT (*p* = 0.015), with no change in acute treatment toxicities [49].

#### 3.2.3. Hypofractionation

MRgRT is also promising in the era of personalized treatments by allowing precise delineation of target and normal tissue volumes for every delivered fraction. This provides an opportunity to investigate and use hypofractionation and stereotactic ablative RT (SABR), which was not previously possible in head and neck cancers due to the presence of highly vulnerable organs in immediate proximity to tumor targets. Hypofractionation is currently being investigated in the phase I DEHART trial, including head and neck cancer patients unfit for chemoradiation who will receive 40 Gy in 15 fractions to elective low-risk regions and 50–60 Gy in 15 fractions to the GTV, with MR-guided volume adaptation at fractions 6 and 11, followed by adjuvant immunotherapy [50]. SHORT-OPC is an ongoing phase II randomized trial for HPV-associated OPC patients, who will receive either standard chemoradiation over 7 weeks or 40 Gy in 20 fractions to elective regions with a SABR boost of 14 Gy in 2 fractions to the GTV. The study is investigating the LRC, toxicity, OS, progression-free survival (PFS) and QoL of this novel technique [51].

## 4. Assessment of Response

As previously mentioned, MRI also has the advantage of providing functional information on tumor and normal tissue behavior, allowing for potential MRI-derived predictive and prognostic biomarkers. Functional MRI techniques include DWI, 3D DCE MRI and spectroscopy, although DWI is the most extensively studied for the assessment of tumor behavior in head and neck cancers.

### 4.1. Before Treatments

The possibility of predicting which patients are at higher risk of incomplete response or treatment resistance offers a great opportunity for treatment intensification. Kim et al. investigated the value of DWI before, during, and 2 weeks after chemoradiation for head and neck cancer and reported significantly lower pre-treatment ADC values in complete responders compared to partial responders [52]. A higher ADC was also associated with local failure on multivariate analysis in a retrospective study by Hatakenaka et al. [53]. The previously mentioned DWI-guided dose-painting phase II trial by Fu et al. is an effective example of how such functional information can be used to tailor treatments and improve oncological outcomes [49]. Although DWI is currently the most studied functional MRI sequence, other baseline MRI radiomics features also show promising results in predicting recurrence and survival outcomes in head and neck cancer patients [54]. A retrospective study by Shukla-Dave et al. showed that the K^trans^ parameter (volume transfer constant) on pre-treatment DCE-MRI was a strong predictor of PFS and OS in stage IV head and neck cancers treated with chemoradiotherapy [55].

### 4.2. During Treatments

Frequent high-quality imaging during treatment with MRgRT uncovers previously unavailable functional information on tumor response that is promising for the purpose of treatment tailoring and adaptation. A systematic review of functional imaging during (chemo)radiotherapy for response prediction in head and neck cancers showed that an increase in mean ADC 3 weeks after treatment commencement predicted significantly better locoregional control [56]. The previously cited INSIGHT-2 trial, which investigates the feasibility of dose escalation at week 2 per response on DWI for HPV-negative OPC patients, is another useful example of how functional imaging can be integrated into treatment planning and adaptation [47].

### 4.3. After Treatments

A retrospective study by Schouten et al. assessing the sensitivity and specificity of DWI and FDG-PET in detecting residual nodal disease after chemoradiation also showed that the addition of DWI to a 3 months post-treatment FDG-PET increased its specificity from 84% to 95%, thereby lowering the risk of unnecessary salvage neck dissections [57]. These results were corroborated by a second small retrospective study showing that a 2-month post-treatment DWI could be equally sensitive and more specific than a 3-month post-treatment FDG-PET in detecting persistent neck disease following chemoradiation for OPC [58]. Another retrospective histopathological study comparing DWI and FDG-PET found that MRI had a higher sensitivity for the detection of local perineural spread and muscle infiltration in residual or recurrent tumors, and better correlates the lesion size to the histopathologic specimen [10]. Furthermore, in the recurrent setting, a systematic review showed better specificity and negative predictive value for DWI compared to FDG-PET for the detection of recurrences from head and neck cancers, which is particularly important to avoid unnecessary biopsies in fragile irradiated tissues [59].

Finally, the vast amount of rich data obtained from numerous high-quality anatomical and functional imaging during and after treatments may provide a unique opportunity to exploit machine learning and artificial intelligence, possibly uncovering new predictive and prognostic biomarkers and improving treatment individualization of head and neck cancer patients [60,61].

## 5. Current Challenges and Limitations

Despite the many benefits and promising emerging avenues associated with the use of MRgRT for head and neck cancers, several technical and clinical limitations are currently hindering its broader use. These include a lack of standardization in terms of acquisition and imaging protocols specific to head and neck imaging. Although the current standard for a diagnostic MRI is a 3 Tesla (T) magnet, the two currently available MR-Linac machines offer 0.35 T and 1.5 T magnets, respectively. The 0.35 T magnet offers many benefits, such as higher frames per second, faster tissue tracking, and lower electron return effect, but it is yet to be determined if it will offer sufficient image quality to translate in clinically impactful effects for head and neck cancer patients [62].

Although the vast amount of information obtained from repetitive imaging during RT treatments may be interesting for treatment individualization, the interpretation and clinical significance of per-treatment changes remain a challenge. Moreover, the integration of MRgRT data in target delineation and treatment adaptation requires more extensive training in MRI interpretation for radiation oncologists, especially with the increasing use of functional MRI sequences. More evidence-based MRI-specific contouring guidelines could also help address this issue [34].

The safe combination of the magnetic field of an MRI with a linear accelerator emitting charged particles has also been an imposing technical challenge. This combination gives rise to a phenomenon known as the electron return effect, caused by the influence of the magnetic field on secondary electrons, and resulting in hot and cold dose spots at interfaces between high- and low-density regions. Fortunately, these can be predicted and integrated in the plan optimization, although variations in the air-tissue interfaces during RT in the head and neck region might put these patients at risk of unplanned dose heterogeneities [63]. Additional shielding on the MR-Linac is also needed to compensate for the mutually detrimental effects of the linear accelerator components and the magnetic field of the MRI, adding significant complexity to gantry design. The complexity and added weight of the gantry preclude simultaneous rotation and treatment delivery, currently limiting its application to static IMRT [62]. This is a particularly important barrier to the adoption of MRgRT in head and neck cancers, as the now standard volumetric modulated arc therapy (VMAT) is associated with better sparing of critical organs at risk such as the brainstem, parotid, esophagus, and oral cavity [64].

It must also be noted that MRI has several contraindications and limitations that preclude several patients from the benefits of MRgRT, including incompatible cardiac implantable electronic devices, metallic implants, and severe claustrophobia [65]. Furthermore, the inability to deliver VMAT, the reduced dose rate due to a larger source-isocenter distance and the time needed for online adaptation limit current MR-Linac platforms to longer treatment durations compared to standard linear accelerator treatments [63]. This prolonged treatment time combined with the loud and enclosed MRI may lead to patient compliance problems, especially considering the 6 to 7 weeks of daily treatments currently required for head and neck cancer, and could also reduce the number of patients treated daily. The integration of adaptation and treatment replanning also requires an increased clinical workflow for the radiation oncologists, the physicists and the department overall, thereby increasing treatment costs. Conversely, this initial increase in workflow could eventually be offset by the further development of hypofractionation and SABR, which would allow shorter treatment courses and potentially reduced costs [66]. The benefit of an MR-Linac compared to repeated offline simulation MRIs after sufficient tumor response also remains unclear, as daily imaging is unlikely to impact outcomes for head and neck cancers, where intra- and inter-fraction motion is relatively limited compared to other disease sites [67]. Adequate cost-effectiveness analyses are therefore critical to weight in the numerous current and future potentials of MRgRT for head and neck cancers against the initial increase in treatment time and clinical workflow.

## 6. Conclusions

With its enhanced soft tissue contrast and functional techniques, MRI has proven to be an excellent tool and has cemented its place in the standard of care for the treatment planning of head and neck cancer patients, allowing for optimal baseline evaluation of tumor local extension and lymph node involvement. The next promising and upcoming roles of MRI for head and neck RT include empowering adaptive RT, increasing the role of hypofractionation, and developing the use of pre-, per- and post-treatment anatomical and functional assessment to inform therapeutic decisions and provide individualized treatments. A few challenges are currently hindering the broader implementation of MRgRT, including a lack of standardization in imaging acquisition, the need for physician education, the risk of dose heterogeneity due to the electron return effect, the current incompatibility with VMAT and the longer treatment duration. Fortunately, several clinical trials are ongoing to justify the cost and associated clinical workflow and to ensure the safety of these novel approaches. Although several trials described in this review are still at the early recruiting stage, they highlight the many promising potentials of MRgRT for head and neck cancer patients. They also provide anticipation that more personalized treatments are forthcoming to improve tolerance and disease control for this heterogeneous patient population that continues to be at risk of the dual perils of severe toxicity from treatment and treatment failure.
